# Metabolomic Biomarkers in Serum and Urine in Women with Preeclampsia

**DOI:** 10.1371/journal.pone.0091923

**Published:** 2014-03-17

**Authors:** Marie Austdal, Ragnhild Bergene Skråstad, Astrid Solberg Gundersen, Rigmor Austgulen, Ann-Charlotte Iversen, Tone Frost Bathen

**Affiliations:** 1 Department of Circulation and Medical Imaging, Faculty of Medicine, Norwegian University of Science and Technology (NTNU), Trondheim, Norway; 2 Liaison Committee between the Central Norway Regional Health Authority (RHA) and Norwegian University of Science and Technology (NTNU), Trondheim, Norway; 3 St. Olavs Hospital, Trondheim University Hospital, Trondheim, Norway; 4 Department of Laboratory Medicine, Children's and Women's Health, Faculty of Medicine, Norwegian University of Science and Technology (NTNU), Trondheim, Norway; 5 National Center for Fetal Medicine, St. Olavs Hospital, Trondheim, Norway; 6 Department of Cancer Research and Molecular Medicine, Faculty of Medicine, Norwegian University of Science and Technology (NTNU), Trondheim, Norway; 7 Centre of Molecular Inflammation Research (CEMIR), Faculty of Medicine, NTNU, Trondheim, Norway; Instituto de Investigación Sanitaria INCLIVA, Spain

## Abstract

**Objective:**

To explore the potential of magnetic resonance (MR) metabolomics for study of preeclampsia, for improved phenotyping and elucidating potential clues to etiology and pathogenesis.

**Methods:**

Urine and serum samples from pregnant women with preeclampsia (n = 10), normal pregnancies (n = 10) and non-pregnant women (n = 10) matched by age and gestational age were analyzed with MR spectroscopy and subjected to multivariate analysis. Metabolites were then quantified and compared between groups.

**Results:**

Urine and serum samples revealed clear differences between women with preeclampsia and both control groups (normal pregnant and non-pregnant women). Nine urine metabolites were significantly different between preeclampsia and the normal pregnant group. Urine samples from women with early onset preeclampsia clustered together in the multivariate analysis. The preeclampsia serum spectra showed higher levels of low and very-low density lipoproteins and lower levels of high-density lipoproteins when compared to both non-pregnant and normal pregnant women.

**Conclusion:**

The MR determined metabolic profiles in urine and serum from women with preeclampsia are clearly different from normal pregnant women. The observed differences represent a potential to examine mechanisms underlying different preeclampsia phenotypes in urine and serum samples in larger studies. In addition, similarities between preeclampsia and cardiovascular disease in metabolomics are demonstrated.

## Introduction

Preeclampsia (PE) is a complex syndrome affecting about 3% of pregnancies [1]. It presents serious risk of both maternal and fetal morbidity and mortality [2]. PE is characterized by high blood pressure and proteinuria in the second half of pregnancy [3]. No tests accurately predict the onset of PE, and implementation of fetal delivery is the only definitive treatment for threatening manifestations of symptoms [1].

The pathogenesis of PE is still undefined. However, it is generally assumed that it starts in early pregnancy with poorly developed placental vascularization, giving rise to placental oxidative stress and imbalanced interaction between maternal and fetal cells. Later, inappropriate and exaggerated maternal responses to the placental stress are established, involving endothelial activation and systemic inflammation [4]. The inflammation in PE shows a strong similarity to the development of cardiovascular diseases (CVD) [4], and it has been reported that women with preeclamptic pregnancies have an up to eight-fold increased risk of later cardiovascular events [5]. The shared underlying mechanisms include endothelial dysfunction, metabolic abnormalities and increased oxidative stress [6].

Metabolites are constituents of the metabolism, chemical interactions in the body necessary for life [7]. Metabolomics is the systematic study of metabolites in tissues and biofluids [8]. The concentrations of metabolites and their combinations can be used as predictive models for disease classification and progression [8]. Robust statistical methods are applied to handle the massive data outputs.

Metabolomics analysis holds potential for detailed phenotyping of the PE syndrome, but few metabolomics studies of women with active disease have so far been undertaken. Studies by Turner *et al*. [9], [10], using MR metabolomics on serum from women diagnosed with PE reported metabolic patterns attributed to oxidative stress including decreased lipid and ketone body content, and the findings indicated that this method could be useful for PE phenotyping. Schott *et al*. [11] used both proton and phosphorous MR spectroscopy to analyze plasma from women with PE. They found a decrease in HDL and a trend towards higher levels of VLDL2 and LDL2 in this group compared to healthy pregnancies. However, no metabolomics studies have analyzed both urine and serum from the same group of women, which represents a more comprehensive view of the metabolome. Detailed analysis of body fluids from women with preeclampsia could improve diagnostic accuracy and possibly predict perinatal outcomes and future risk for the mother.

Further research is needed to establish the role of metabolomics and the robustness of the method in preeclampsia. Furthermore, a discussion of the discriminatory metabolites in a more biological context relevant to PE is generally lacking. To this end, the aim of the present study was to establish the metabolic profiles of body fluids (urine and serum) from women with PE, normal pregnancies and from non-pregnant women by MR metabolomics. Detailed phenotyping of the PE syndrome and potential clues to etiology and pathogenesis were explored.

## Materials and Methods

### Ethics Statement

All participating women signed informed consents and the study was approved by the Regional Committee for Medical and Health Research Ethics (REC), Central Norway, reference number 2011/761.

### Study Population

Women admitted with PE at the maternity ward at St. Olavs Hospital, Trondheim University Hospital, Norway gave samples to the study. The PE diagnosis was based on the diagnostic criteria of the Norwegian Medical Association (blood pressure ≥ 140/90, proteinuria ≥+1, measured at least twice four to six hours apart after gestational week 20) [12]. Pregnant and non-pregnant control women were recruited by appeals to environments of St. Olavs Hospital and Røros Medical Center, Røros, Norway. Control women with previous PE pregnancies were not included. Gestational age for both cases and controls were based on routine ultrasound examination between gestational week 17 and 20. Information about health status and pregnancy was collected from interviews and medical journals. All included women were of Scandinavian ethnicity.

### Sample Handling and Spectroscopy

Peripheral venous blood (5 mL) and spot urine samples (20 mL) were collected from nonfasting women with PE at time of diagnosis, from healthy pregnant women matched by age and gestational age to the PE group, and from non-pregnant women matched by age to the PE group. Aliquots (1.8 mL) were stored at −80°C prior to analysis.

Samples were thawed at 20°C, mixed with bacteriostatic buffer and stored at 5°C until analysis (≤15 hours). Urine was centrifuged at 6000 RPM (Sorvall RMC 14; DuPont) for five minutes. The supernatant (540 μL) was mixed with buffer (60 μL) (pH 7.4 1.5 mM KH_2_PO_4_ in D_2_O, 0.1% Trimethyl-Silyl Propionate (TSP), 2 mM NaN_3_) and analyzed in 5 mm NMR tubes (Norell Inc., NJ, USA). Serum (100 μL) was mixed with buffer (100 μL) (pH 7.4 0.075 mM Na_2_HPO_4_, 5 μM NaN_3_, 5 μM TSP) and analyzed in 3 mm NMR tubes. MR analysis was performed at the MR Core Facility at NTNU, Trondheim, Norway using a Bruker Avance III Ultrashielded Plus 600 MHz spectrometer (Bruker Biospin GmbH, Germany) equipped with 5 mm QCI Cryoprobe with integrated, cooled preamplifiers for ^1^H, ^2^H and ^13^C. Experiments were fully automated using the SampleJet™ in combination with Icon-NMR on TopSpin 3.1 software (Bruker Biospin). Proton spectra were acquired using 1D NOESY with presaturation and spoil gradients on urine samples and a 1D lipid and water suppressing Carr-Purcell-Meiboom-Gill sequence (CPMG) on serum samples. Diffusion edited serum spectra (LEDBPG) for suppression of small molecular weight metabolite signals were also acquired. Additional 2D spectra of urine samples were acquired for metabolite identification: J-resolved spectroscopy (JRES), Heteronuclear Multiple Bond Correlation Spectroscopy (HMBC), Heteronuclear Single Quantum Coherence Spectroscopy (HSQC) and Total Correlation Spectroscopy (TOCSY). Additional NMR parameters are given in Tab 3. Spectra were Fourier transformed to 128 K after 0.3 Hz exponential line broadening. Chemical shifts were referenced to TSP (δ0 ppm).

### Multivariate analysis

In MR Metabolomics, common statistical methods are Principal Component Analysis (PCA) and Partial Least Squares Discriminant Analysis (PLS-DA) [13]. PCA is a powerful method of data extraction, which finds combinations of variables that describe trends in large data, called principal components (PCs), visualized in scores and loading plots. The score plots show each spectrum as an object in the principal component space, and are useful for identifying clusters and outliers in the dataset. The loading plots show the contributing variables to each PC. PLS-DA models the relationship between the spectra and class information using multivariate regression methods. The metabolites responsible for the separation between classes are shown in loading variables (LVs), and may be colored by variable importance in projection (VIP) [13]. Multivariate analysis was performed using PLS_Toolbox 6.7.1 (Eigenvector Research, USA).

Spectra were imported to Matlab r2012a (The Mathworks, Inc., MA, USA). Residual water signals were removed. The urine NOESY spectra were normalized to equal area below the curve, cut to region of interest (ROI) (δ8–1 ppm) and peak aligned using *i*coshift [14]. The serum CPMG spectra were cut to ROI (δ4.5–0.5 ppm) and aligned by referencing the left alanine peak at δ1.50 ppm. The serum LEDBPG spectrum were cut to ROI (δ1.45–0.77 ppm) containing signals from methyl and methylene groups from lipoproteins, and normalized to equal area for additional analysis of the lipid profile.

Spectra sets were mean centered and explored by PCA with random subset cross validation for initial visualization of the data and detection of inherent trends and outliers. Using PLS-DA, a classification model was created on samples from the women with preeclampsia and healthy pregnant groups using the number of LVs giving the smallest classification error, and cross validated by “leave one out” which creates the model on all but one sample, testing it on the remaining sample. Permutation testing with 1000 repeats (random reshuffling of classes, then creating a new predictive model) was done to measure the significance of the predictive model at 95% compared to a classification in arbitrary groups.

### Identification and quantification of metabolites

Metabolites were assigned using Bruker AMIX software v.2.5 (Analysis of MIXtures software, Bruker Biospin) and Chenomx v.7.11 (Chenomx Inc., Alberta, Canada), matching spectra to reference databases of metabolites. 2D NMR spectra (JRES, COSY, HSQC, and HMBC) were reviewed to confirm assignments. Additional assignments were done with help from literature [7], [15]. All identified metabolites were quantified in Chenomx, based on the visible TSP concentration. TSP was quantified in Topspin using the PULCON [16] principle based on a creatine (14.43 mM) spectrum recorded at equal parameters. Serum metabolites were quantified from CPMG spectra, and urine metabolites from NOESY spectra. Concentrations were imported to SPSS v. 20.0.0 (IBM Corp, NY, USA) and subjected to Kruskal-Wallis test of three independent samples. Urine metabolite concentrations were analyzed as [metabolite/creatinine] ratio to correct for dilution. The significance cutoff was set to p<0.05 after Benjamini-Hochberg correction [17] of p-value for multiple parallel tests.

## Results

Details of the study groups are given in Table 1. Ten women with PE, ten healthy pregnant women and ten non-pregnant women were included. Age and gestational age were matched, and in accordance with the PE diagnosis, proteinuria and blood pressure were significantly different between the two pregnant groups (not measured in the nonpregnant group).

**Table 1:** Characteristics of study participants.

Data	PE	PC	NP	p-value
n (samples)	10	10	10	-
Age (years)	29.5 (22–39)	32.6 (28–39)	30.7 (24–39)	>0.05
GA at sampling (week)	35.9 (21.7–37.9)	35.2 (18.6–37.4)	N/A	>0.05
GA at onset (week)	33.2 (21.4–36.9)	N/A	N/A	-
BP sys. (mmHg)	163 (143–174)	120(100–191)	N/A	0.000
BP dia. (mmHg)	104 (96–111)	78 (60–96)	N/A	0.000
Proteinuria^a^	3 (1–4)	0.1 (0–1)	N/A	0.000

*Values are given as median (min-max). PE: Women with preeclampsia. PC: Pregnant controls. NP: Non-pregnant controls. GA: Gestational age. BP: Blood pressure. Dia: Diastolic. Sys: Systolic. N/A: Not applicable. Statistical p-values computed by Kruskal-Wallis independent samples test.*

a *Proteinuria measured with dipstick.*

**Figure 1:**
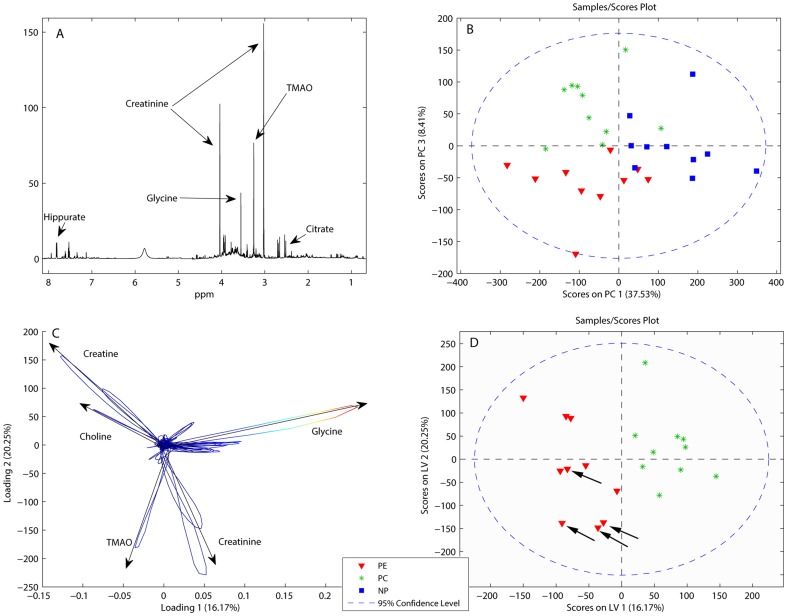
Results from urine analysis. Results from Principal Component Analysis (PCA) and Partial Least Squares Discriminant Analysis (PLS-DA) of urine samples from women with preeclampsia (PE, pregnant controls (PC) and non-pregnant controls (NP). **A**) Typical high resolution NMR spectrum of urine from a PE subject, most abundant metabolites annotated. **B**) PCA score plot separating all three groups in two dimensions. **C**) Loading Variables (LV) 1 and 2 of the PLS-DA used to create a model discriminating between PE and PC groups. Arrow direction indicates increased metabolite level. **D**) Scores on LV1 and LV2 showing a clustering of early onset PE samples (marked by arrows). TMAO: Trimethylamine-N-Oxide.

Results from the urine analyses are shown in Figure 1 and Table 2, with a typical urine spectrum shown in Figure 1A. The urine PCA (Figure 1B) score plot shows clustering of the three groups. PC1 separated samples from both the PE and healthy pregnant groups from the non-pregnant group based on a combination of higher creatinine and trimethylamine-N-oxide (TMAO) levels, and lower glycine levels for the non-pregnant group. PC3 separated preeclamptic women from healthy pregnant women similarly to the subsequent PLS-DA analysis. Creatinine levels were similar between preeclamptic and healthy pregnant women. The PLS-DA model classified urine spectra from preeclamptic and healthy pregnant groups with 95% accuracy (sensitivity = 0.9 and specificity = 1.0) using two LVs (Figure 1C and 1D) and was significant at p≤ 0.001. The model separated the groups based on a combination of higher choline and creatine levels, and lower glycine levels for preeclamptic women compared to healthy pregnant women. Urine samples from women with early onset PE (<34 weeks) had lower scores on LV2 than the late onset women. (Fig 1D, starred). These had higher TMAO and creatinine, and lower choline and creatine compared to the late onset PE group. The urinary metabolite concentrations are shown in Table 2. The variation in concentrations was similar to the results from the multivariate analyses. Twenty-one metabolites were significantly different between all three groups at p<0.05, with nine metabolite concentrations significantly different between women with PE and healthy pregnant women, and 15 between healthy pregnant women and non-pregnant women. In summary, urine sample spectra from the PE group were clearly different from those of healthy pregnant women based on metabolite content, and a difference in metabolic profile between women with early and late onset PE may exist. Healthy pregnant women also showed a different urinary metabolic profile than non-pregnant women, with higher excretion of amino acids.

**Table 2:** Urine metabolite concentrations.

Metabolite (μM/mM creatinine)	PE	PC	NP	p-value
Glycine* †	260±150	498±219	138±79	0.000
p-Cresol Sulfate*	6.2±4,3	39±13	30±10	0.000
Alanine†	67±57	86±51	21±5	0.000
Threonine†	84±102	94±48	20±5	0.000
Choline†	41±50	10±4	5.3±2.7	0.004
Hippurate*	88±61	265±144	280±188	0.004
Histidine* †	153±129	266±100	79±42	0.004
Asparagine* †	31±42	47±24	13±7	0.004
Isobutyrate* †	21±8	13±7	6±7	0.004
Lactate†	36±27	73±137	9.6±4.4	0.004
Citrate	203±107	473±136	371±217	0.004
Leucine†	9.4±4.8	8.0±1.6	5.0±1.3	0.010
Dimethylamine*	66±13	41±7	40±18	0.015
Trigonelline*	6.3±4.9	16±15	29±22	0.015
2-Oxoglutarate	36±23	40±9	13±10	0.016
Ethanolamine†	75±24	66±19	44±12	0.018
Isoleucine†	6.3±3.8	5.2±1.2	3.1±1.1	0.018
cis-Aconitate†	37±15	39±12	25±5	0.018
Creatine	131±118	76±138	23±25	0.041
Glutamine†	114±88	121±37	70±20	0.041
Glucose* †	38±71	75±44	35± 15	0.049
Valine†	7.6±6.7	6.1±2.0	3.8±1.4	>0.05
Tyrosine	28±19	23±12	14±6	>0.05
N-N-Dimethylglycine	6.0±4.0	5.3±3.0	3.3±1.7	>0.05
Malonate	58±49	156±187	72±105	>0.05
Uracil	8.0±3.8	10.2±2.8	7.1±3.2	>0.05
N-Phenylacetylglycine^a^	41±29	71±35	57±24	>0.05
Betaine	12±8	15±13	8.0±5.2	>0.05
2-Methylglutarate	11±7	16±9	6.9±3.2	>0.05
Guanidoacetate	70±77	77±41	63±39	>0.05
Formate	23±12	35±18	23±13	>0.05
3-Hydroxybutyrate	9.3±7.9	16±15	16±25	>0.05
Acetate	8.5±5.8	11±5	12±9	>0.05
Urea	2957±1211	2251±1077	2795±1845	>0.05
2-Hydroxybutyrate	8.0±3.5	7.4±1.2	5.9±1.3	>0.05
Pyruvate	7.8±4.9	15±15	6.3±2.1	>0.05
TMAO	67±58	54±54	66±78	>0.05
Creatinine^b^	30 ±38	21±13	26±14	>0.05
o-Acetylcholine	2.1±3.1	1.7±2.1	1.4±0.8	>0.05
π-Methylhistidine	46±55	48±67	50±56	>0.05
Phenylalanine	17±13	15±8	14±5	>0.05

*Values given as mean [metabolite/creatinine]±sample standard deviation. PE: Women with preeclampsia. PC: Pregnant controls. NP: Non-pregnant controls. TMAO: Trimethylamine-N-Oxide.*

a *As suggested by Chenomx, may instead be phenylacetylglutamine.*

b *Absolute creatinine concentration – not corrected for dilution.*

*Significantly different metabolite concentration between PE and PC with a cutoff value at p = 0.05 after Benjamini-Hochberg correction using the Kruskal-Wallis test for nonparametric distributions of concentrations for three independent groups.

† *Significantly different metabolite concentration between PC and NP.*

**Figure 2:**
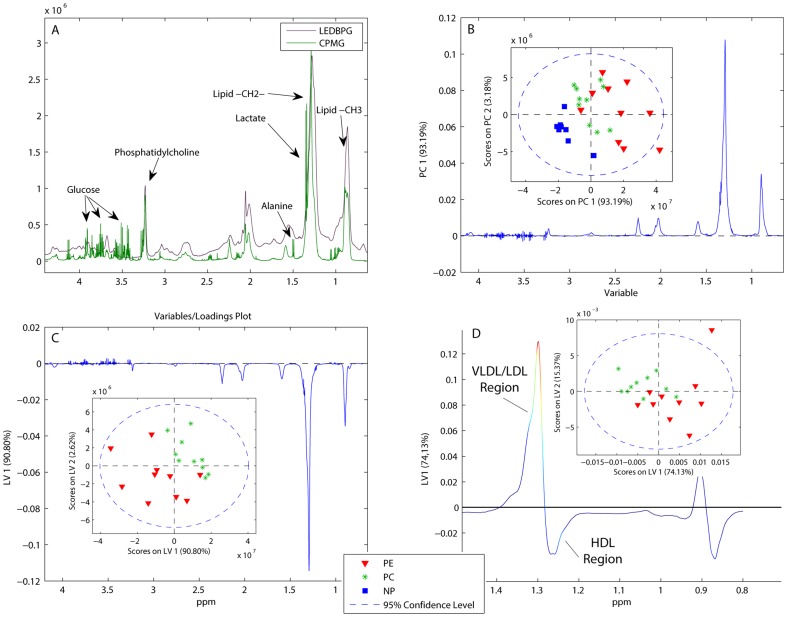
Results from serum analysis. Results from Principal Component Analysis (PCA) and Partial Least Squares Discriminant Analysis (PLS-DA) on the spectra of serum samples from women with preeclampsia (PE), pregnant controls (PC) and non-pregnant controls (NP). ppm: parts per million, resonance frequency of metabolite. **A**) Typical highly resolved serum CPMG (lipids suppressed) and LEDBPG (small metabolites suppressed) spectra from a woman with PE with some annotated metabolites. **B**) Scores plot and loading profile of the PCA separating CPMG spectra of the three groups. **C**) Scores plot and Loading Variable (LV) 1 from the PLS-DA of CPMG spectra showing class discrimination based on lipid level, where women with PE clearly have higher levels of total lipids in the serum compared to pregnant controls **D**) Score plot and LV1 of the LEDBPG showing distinction between PE and PC groups based on lipoprotein distribution. LV1 shows higher levels of VLDL-LDL and lower levels of HDL. VLDL: very low density lipoproteins. LDL: low density lipoproteins. HDL: high density lipoproteins.

Results from the serum analyses are shown in Figure 2 and Table 3, with a typical serum spectrum in Figure 2A. The CPMG and LEDBPG spectra of all serum samples were explored using PCA. A trend of metabolite profiles showing a continuous change from non-pregnant women through healthy pregnant women to women with PE was found, mainly based on increasing total serum lipid content (Figure 2B). All pregnant women had higher serum lipid content than the non-pregnant women, and women with PE had even higher serum lipid content. The distribution of lipoproteins was also different between groups, with the PE group expressing higher signals originating from VLDL and LDL and lower signals from HDL. The signals from the lipoproteins in the NMR spectra consist of several highly overlapping peaks, arising from the lipid moieties within the various lipoproteins. The chemical shifts differ slightly between the particles due to the density differences of the lipoproteins, with lower densities at higher chemical shifts (Figure 2D) [18]. PLS-DA classified serum CPMG spectra from women with PE and healthy pregnant women with 90% accuracy (sensitivity = 0.8 and specificity = 1.0) using four LVs, with heavy reliance on lipid levels for separation between PE and PC (Figure 2C). The lipoprotein distribution was explored further in a PLS-DA on the LEDBPG spectra, as shown in Figure 2D. PE cases were discriminated from healthy pregnant controls by the lipoprotein profile alone, with increased signal in the LDL-VLDL region (the leftmost part of the lipid signal) and decreased signal in the HDL region of the serum spectra. Visible serum metabolites concentrations are shown in Table 3. Serum from women with PE had significantly lower concentrations of histidine than the healthy pregnant women, and non-significant lower levels of formate and higher levels of glycerol. Healthy pregnant women had higher alanine and lactate than the non-pregnant women.

**Table 3:** Serum metabolite concentrations.

Metabolite (μM)	PE	PC	NP	p-value
Histidine*	90±26	72±21	57±12	0.036
Formate	19±4	25±4	22±2	0.059
Glycerol	146±37	105±34	92±29	0.059
Alanine†	251±39	303±82	223±31	0.059
Lactate†	907±306	1094±252	774±179	0.094
Creatine	42±14	30±7	35±7	>0.05
Glucose	2455±623	3005±656	2922±466	>0.05
Glycine	135±31	142±20	176±52	>0.05
Valine	132±26	155±31	161±26	>0.05
Acetate	22±4	26±9	32±16	>0.05
Citrate	81±18	70±19	63±13	>0.05
Phenylalanine	38±7	37±10	34±4	>0.05
Glutamine	366±95	375±81	410±60	>0.05
3-Hydroxybutyrate	80±58	47±13	51±34	>0.05
Tyrosine	34±8	39±8	39±10	>0.05
Glutamate	94±28	106±35	95±37	>0.05
Creatinine	54±6	50±7	53±7	>0.05
Leucine	116±18	114±28	109±25	>0.05
2-Methylglutarate	11±3	12±2	11±4	>0.05

*Values given as mean±SD. PE: Women with preeclampsia. PC: Pregnant controls. NP: Non-pregnant controls.*

*Significantly different metabolite concentration between PE and PC with a cutoff value at p = 0.05 after Benjamini-Hochberg correction using the Kruskal-Wallis test for nonparametric distributions of concentrations for three independent groups.

† *Significantly different metabolite concentration between PC and NP.*

## Discussion

The present study clearly demonstrates the metabolic differences between the women with PE and those with healthy pregnancies, in both urine and in serum. The metabolomics method additionally reveals a possible way to subgroup the disease based on metabolic profiles. The metabolic profiles gave new information about possible pregnancy- and disease-induced changes.

The strength of multivariate metabolomic analysis is that the entire visible metabolome is taken into account; and metabolites with small and large variation contribute to the end result. As a result there are limitations towards finding definite mechanisms correlating to alterations in isolated metabolites. Many small metabolites visible to MR metabolomics are involved in several pathways, and may not be comparable between the urine and serum metabolome.

Differences associated with normal pregnancy compared to non-pregnant women included increased amino acids, choline, and lactate in urine, and higher alanine and lactate in serum. A similar pattern was found in a study by Diaz *et al* [19] following healthy pregnancies with MR metabolomics on urine. The increased excretion of amino acids is suggested to be caused by impaired renal filtration in healthy pregnancies [19]. The choline increase appears as a trend increasing from non-pregnant to healthy pregnant to preeclamptic women. Although choline metabolism appears important to fetal development [20], it is difficult to pinpoint the exact cause of the increase. The increase of lactate in both urine and serum from pregnant women confirms the findings by other studies [19], [21], and follows an increase in prolactin linked to lactation [21].

Women with PE showed increased choline and decreased glycine, p-cresol sulfate and hippurate in urine, which may be related to increased oxidative stress and kidney dysfunction. Choline and glycine are connected through the metabolic pathway of homocysteine [22]. A previous study found elevated choline in serum of women with preeclampsia, and connected the findings to this pathway [20]. Reduced glycine has also been seen with preterm birth [20]. Urinary choline increase has been associated with fetal stress in the second trimester [23]. Glycine is a precursor to glutathione, a tripeptide important for protection against oxidative stress [20]. The decrease in glycine excretion in women with PE could be a result of increased demand for glutathione in response to oxidative stress. p-Cresol sulfate is retained in patients with kidney damage [24], and is accordingly reduced in the PE group which suffers from kidney dysfunction. p-Cresol sulfate is known to increase oxidative stress in human kidney epithelial cells [24]. Possible consequences of retained p-cresol sulfate could be further increased kidney damage and systemic inflammation in women with PE, thus contributing to the severity of the disease. Hippurate is a metabolic conjugate of glycine, and may be reduced as a consequence of reduced glycine in PE. A relation between hippurate excretion and PE has not been reported previously. The multivariate analysis grouped the urine samples from women with early onset PE together, indicating a similarity in their metabolic profile compared with women with late onset PE, and a difference in phenotype between the two. Such a division was previously found in serum samples from women in early pregnancy [25], [26]. However, as there were only four women in this group the results should be interpreted with caution and be followed up in a larger study.

Metabolic profiles in maternal serum revealed significant differences with regards to PE. The major difference detected was the higher total serum lipid content and an increase of VLDL/LDL signals for the PE group. Women with PE also had higher histidine and glycerol levels than women with normal pregnancies. Increased histidine levels were in accordance with the study by Bahado-Singh *et al* which looked at first-trimester serum [26]. Bolin *et al* [27] found disturbance in histidine metabolism, with contrarily decreased histidine-rich glycoprotein in serum throughout pregnancies which later develop PE. Although the histidine contained in glycoproteins is different from the free histidine seen in MR spectra, their metabolism may be related. Histidine-rich glycoproteins interact with the coagulation system and angiogenic pathway, and a decrease was found to predict PE in Bolins study [27]. Increased glycerol was detected in the women with PE, similar to the Bahado-Singh study [26], where it was attributed to abnormal lipid metabolism as it forms the backbone of triglycerides. The lipoprotein profiles here shown related to PE are similar to those found for people at risk of CVD [28], with increased low density lipoprotein levels. Lipid dysfunction starts early in pregnancies destined for PE development [28], suggesting that metabolomics may be used to predict the onset of PE. An increase in low- and very-low density lipoprotein has been recorded in patients with CVD and PE previously [28], underscoring the similarities between the two diseases.

The quantification of serum metabolites was done on T2-edited CPMG spectra, where lipid signals are attenuated. Therefore the concentrations of metabolites in serum are not absolute, but comparable between spectra. The multivariate analysis performed on the LEDBPG spectra, where small molecular weight metabolite signals are filtered out, showed that the lipid profile itself was sufficient to distinguish between the two groups.

The study contains relatively few samples, limiting a complete validation procedure. However, a rigorous cross validation was performed to ensure that the model was valid also for samples not included in the building of the model. As this is an exploratory study highlighting main differences between groups, cross validation in combination with permutation testing is sufficient to conclude whether there is a difference between groups. Analysis of spectra using PLS-DA is prone to overfitting. However, permutation testing of the urine and serum PLS-DA models (Table 4) revealed them to be significantly different (p<0.05) from models made on random classifications. This indicates that the spectra contained sufficient information to distinguish between samples from women with PE and healthy pregnant women.

**Table 4:** PLS-DA Classification of samples as healthy pregnant or from women with preeclampsia.

Input	LVs	Classification accuracy	Sensitivity	Specificity	AUC	p
Urine spectra	2	95%	0.9	1	0.90	0.001
Serum CPMG spectra	4	90%	0.8	1	0.86	0.002
Serum LEDBPG spectra	4	90%	1	0.8	0.70	0.001

The sensitivity is for detecting a preeclampsia sample using Partial Least Squares Discriminant Analysis. Classification accuracy, sensitivity and specificity are from the leave-one-out cross validation. The p-value is from permutation testing the model with 1000 repeats. LVs: number of loading variables in model. AUC: Area under the Receiver Operator Characteristic curve. CPMG: Lipid-suppressed. LEDBPG: Low molecular weight metabolite suppressed.

The metabolic changes found in cases compared to controls reflect the disease state of the individual. However, it is possible that some of the changes may be evident in the metabolome before the onset of the disease. This possibility must be evaluated in longitudinal studies following women earlier in their pregnancies.

Urine and serum from women with PE, normal pregnancies and non-pregnant women were effectively discriminated by MR metabolomics. Differences were observed related to disease processes and phenotypes. Samples from healthy pregnancies were clearly different from samples collected from non-pregnant women. The observed data suggest that an enlarged study is recommended to find predictive biomarkers earlier in pregnancy or to sub-phenotype the disease, and to investigate the association to later cardiovascular symptoms.
